# Neuroinflammatory and Metabolomic Temporal Dynamics Following Wood Smoke Inhalation

**DOI:** 10.21203/rs.3.rs-3002040/v1

**Published:** 2023-06-05

**Authors:** David Scieszka, Yan Jin, Shahani Noor, Ed Barr, Marcus Garcia, Jessica Begay, Guy Herbert, Russell P Hunter, Kiran Bhaskar, Rahul Kumar, Rama Gullapalli, Alicia Bolt, Mark A. McCormick, Barry Bleske, Haiwei Gu, Matthew Campen

**Affiliations:** University of New Mexico, College of Pharmacy; Florida International University, Center for Translational Sciences; University of New Mexico, Department of Molecular Genetics and Microbiology; University of New Mexico, College of Pharmacy; University of New Mexico, College of Pharmacy; University of New Mexico, College of Pharmacy; University of New Mexico, College of Pharmacy; University of New Mexico, College of Pharmacy; University of New Mexico, Department of Molecular Genetics and Microbiology; University of New Mexico, Department of Pathology; University of New Mexico, Department of Pathology; University of New Mexico, College of Pharmacy; University of New Mexico, Department of Biochemistry and Molecular Biology; University of New Mexico, Department of Pharmacy Practice and Administrative Science; Florida International University, Center for Translational Sciences; University of New Mexico, College of Pharmacy

**Keywords:** Particulate matter, wildfire smoke, blood brain barrier, neuroinflammation, inhalation

## Abstract

Smoke from wildland fires has been shown to produce neuroinflammation in preclinical models, characterized by neural infiltrations of neutrophils and monocytes, as well as altered neurovascular endothelial phenotypes. To address the longevity of such outcomes, the present study examined the neuroinflammatory and metabolomic temporal dynamics after inhalation exposures from biomass-derived smoke. 2-month-old female C57BL/6J mice were exposed to wood smoke every other day for two weeks at an average exposure concentration of 0.5mg/m^3^. Subsequent serial euthanasia occurred at 1-, 3-, 7-, 14-, and 28-days post-exposure. Flow cytometry of right hemispheres revealed two endothelial populations of PECAM (CD31), high and medium expressors, with wood smoke inhalation causing an increased proportion of PECAM^Hi^. These populations of PECAM^Hi^ and PECAM^Med^ were associated with an anti-inflammatory and pro-inflammatory response, respectively, and their inflammatory profiles were largely resolved by the 28-day mark. However, activated microglial populations (CD11b^+^/CD45^low^) remained higher in wood smoke-exposed mice than controls at day 28. Infiltrating neutrophil populations decreased to levels below controls by day 28. However, the MHC-II expression of the peripheral immune infiltrate remained high, and the population of neutrophils retained an increased expression of CD45, Ly6C, and MHC-II. Utilizing an unbiased approach examining the metabolomic alterations, we observed notable hippocampal perturbations in neurotransmitter and signaling molecules like glutamate, quinolinic acid, and 5-α-dihydroprogesterone. Utilizing a targeted panel designed to explore the aging-associated NAD^+^ metabolic pathway, wood smoke exposure drove fluctuations and compensations across the 28-day time course, ending with decreased hippocampal NAD^+^ abundance at day 28. Summarily, these results indicate a highly dynamic neuroinflammatory environment, with potential resolution extending past 28 days, the implications of which may include long-term behavioral changes, systemic and neurological sequalae directly associated wtith wildfire smoke exposure.

## INTRODUCTION

Wildland fire acres burned per year have roughly doubled since 1985[1]. These fires now routinely generate smoke that deteriorates air quality for most of the country. This smoke contains a multifarious mixture of toxic components due to myriad combustion fuel sources (*e.g*., biomass, homes, cars etc.). These wildfire exposures have been linked to cardiovascular[2], pulmonary[2, 3], ocular[4, 5], nasal[5], and recently neurological outcomes[6, 7].

Neuroinflammation following acute and subchronic exposures to wildfire smoke, along with other pollutants like ozone and diesel emissions have been reported[8–11]. The neurological outcomes are postulated to arise from responses to pollutants in the lungs, with previous work positing that inhaled particulate matter (PM) induces pulmonary proteolysis, which in turn generates fragmented peptides that enter the circulation and impair the blood-brain barrier (BBB) integrity through endothelial receptor antagonism[6, 12–15]. Phenotypically, the BBB includes two separate populations of endothelial cells expressing differential levels of platelet endothelial cell adhesion molecule-1 (PECAM or CD31), with a proinflammatory phenotype coinciding with reduced relative PECAM expression[16, 17]. The BBB is a tightly connected system of endothelial cells and pericytes wrapped by astrocytic end-feet, ensuring a regulated separation between peripheral circulation and the central nervous system (CNS). When the lung-derived fragmented peptides arising from pollutant inhalation interact with neurovascular endothelial cells and cause these junctions to fail, current understanding dictates an upregulation of intercellular adhesion molecule-1 (ICAM-1) and chemokine C-C motif ligand 2 (CCL2), with additional downstream activation of resident astrocytes and microglia[12–15]. Previously, a 20-day exposure to naturally occurring wildfire smoke particles altered the distribution of the neurovascular endothelial cell phenotypes, with an increased proportion of PECAM-high (anti-inflammatory) cells exhibiting a reduced level of CCL2, inducible nitric oxide synthase, and tumor necrosis factor-α (TNFα) following particle inhalation[6]. PECAM-medium expressing endothelial cells, however, exhibited higher expression of those same inflammatory proteins, implying that the inflammatory response was mixed and exhibiting a switchover from induction to resolution.

Peripheral immune cells can express leukocyte function-associated antigen-1 (LFA-1) andinteract with CCL2, which both act as transmigration signals for peripheral invasion into the neurological milieu. However, myeloid cells can also express CCL2 in response to stimuli[18]. In addition to CCL2, the vascular cell adhesion molecule-1 (VCAM-1) is recognized by peripheral immune cells for a trans-BBB migration effect, and further mediates peripheral immune invasion[19]. Additionally, neutrophils can express lymphocyte antigen 6 complex (Ly6C), while activated microglia and microglial precursors can express the marker as well[20].

Previous studies have shown that wildfire smoke particulate matter exposure caused only modest pulmonary inflammation yet initiated numerous neuroinflammatory sequelae, including microglial activation, peripheral immune infiltration, and decreased neuroprotective metabolites[6, 7]. To better explore the timing of resolution of this neuroinflammation, we sought to replicate biomass smoke inhalation exposure in a lab setting. Using a serial euthanasia study design, we investigated the onset of inflammatory endothelial response and resolution of inflammation through flow cytometry and metabolomics approaches.

## MATERIAL AND METHODS

### Animals and Exposures

**Figure 1:**
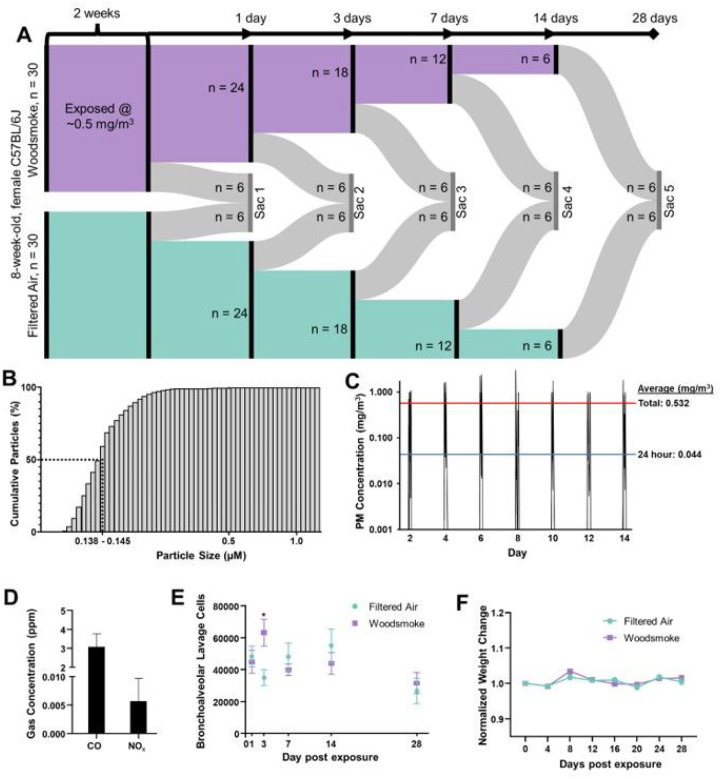
Study Design and Exposure Characterization. (A) 8-week-old female C57BL/6J mice were exposed to woodsmoke derived from woodchip biomass every other day for two weeks at an exposure concentration average ~500μg/m^3^ per exposure. After exposure, mice were euthanized at serial timepoints of 1-, 3-, 7-, 14-, and 28-days post-exposure. (B) Particle size distribution percentile curve. Median particle size measured between 0.138μm-0.145μm. (C) Exposure averaged 532μg/m^3^ for total exposure duration and 0.44 mg/m^3^ for 24-hour average. Red line: exposure average per 4 hours exposure. Blue line: exposure average considering 24 hours per day. (D) Biomass-derived carbon monoxide (CO) and oxides of nitrogen species (NO_x_) concentrations (both below USEPA standards). (E) Bronchoalveolar lavage fluid was extracted for cell staining and differential analyses. Total cells were statistically increased in the lung on day 3, but no other differences were seen for other cells or on other days. (F) No significant weight differences were seen between groups for the duration of experimentation.

Female C57BL/6 mice (Jackson Labs) at 8 weeks of age were housed in AAALAC-approved facilities, on a 12h light:dark cycle and provided a standard chow diet and water ad libitum, in quarantine for one week of acclimation prior to exposure. The exclusive use of female C57BL6/J mice in our study was strategically chosen due to evidence of sex-based differences in inflammatory and metabolic responses, particularly relevant to neuroinflammation[21, 22]. This selection allows us to isolate sex-specific factors influenced by unique hormonal and genetic backgrounds, thereby providing a more refined exploration of the female response. Additionally, our choice helps address a historical bias in research favoring male subjects, and thus fills a significant knowledge gap in female biological processes. Consequently, our decision aligns with both the specific scientific inquiry and broader principles of achieving comprehensive biological understanding. A total of 60 mice were used, randomly and evenly divided into filtered air (FA) control and woodsmoke (WS) exposed groups, with an n = 6 per exposure per time point (Fig. 1). All procedures were conducted with approval by the University of New Mexico Institutional Animal Care and Use Committee. Exposures were conducted in BioSpherix Medium A-Chamber, with mice in reusable shoebox plastic animal case systems with standard wire tops; water was available to mice throughout the exposures, but food was withheld (4h/d). Biomass smoke production was facilitated by a ceramic furnace surrounding a quartz tube connected to a dilution chamber, with subsequent plumbing into the exposure chamber[23]. Approximately 100 mg of piñon wood chips were used per 4h exposure. Smoke exposure was facilitated by vacuum and/or pressurization, with total pressure monitoring to ensure min/max pressure never exceeded −/+ 25mm Hg within the exposure chamber. Concentrations were adjusted manually to ensure a consistent range of WS, using a dump vacuum/pressurizing pump to remove smoke from the exposure chamber or vacuum/pressurizing pumps to channel more smoke from the biomass burning tube into the dilution chamber or reduce flow from the dilution chamber. Mice were exposed whole-body for 4 h every other day for 14 days. We elected this exposure paradigm to mimic intermittent exposure to wood smoke, which aligns more closely with real-world exposure scenarios while mitigating the potential stress of daily exposure. This duration and frequency were also chosen based on previous research demonstrating significant yet non-lethal physiological alterations within similar timelines, allowing us to study the temporal dynamics and resolution of the resultant neuroinflammation and metabolic disturbances[6]. After the last round of exposures, groups of mice were euthanized intermittently over a 28-day period with 6 FA and 6 exposed mice euthanized 1-, 3-, 7-, 14-, and 28-days post-exposure (Fig. 1A–C).

### Particulate Matter Characterization

Exposure concentrations were measured in real-time from a sampling tube positioned centrally in the exposure chamber with a DustTrak II (TSI, Inc; Shoreview, Minnesota) and also gravimetrically using 47 mm quartz filter collected for the duration of each daily exposure and weighed on a microbalance (XPR6UD5, Mettler Toledo) in a temperature-controlled laboratory. Particle size distribution was quantified for the overall system prior to mouse exposures (Laser Aerosol Spectrometer 3340A, TSI), again sampling directly from the center of the exposure chamber. Size distribution was measured over a single 2-hour run (0.138–0.145 μg; Fig. 1B).

### Brain tissue digestion for flow cytometry

Under isoflurane anesthesia, all 12 mice per timepoint underwent transcardial ice-cold 0.1M PBS (pH = 7.4) perfusion. Hippocampus samples were dissected from the left hemisphere for metabolomics analysis (below), and all portions of the left hemisphere were flash-frozen in liquid N2. Right hemispheres from all mice were used for flow cytometry. For flow cytometry, tissues were harvested in ice-cold HBSS buffer and processed immediately according to Miltenyi gentleMACSTM adult neural tissue digestion protocol, as described[6, 24]. Briefly, brain tissues were minced with fine-tip scissors and processed with enzymatic and mechanical digestion steps with gentleMACS TM Octo dissociator with heaters (Miltenyi Biotec, CA, USA). Following digestion steps, cell suspensions were passed through 7μm cell strainers. Myelin debris was removed with Debris Removal Solution (Miltenyi Biotec, CA, USA, 130-109-398), according to the manufacturer’s protocol. Cells were resuspended in PBS (without calcium and magnesium, Sigma-Aldrich, St. Louis, MO) and kept on ice until proceeding to viability dye staining.

### Cell staining for surface and intracellular antigens for flow cytometry

For flow cytometry analysis, live cells were counted on a hemocytometer using trypan blue staining exclusion criteria. Between 0.2—1.0×10^6^ cells were transferred in fluorescence activated cell sorting (FACs) tubes, stained with viability dye eFlour 450 (eBioscience, San Diego, CA) for 30 mins. After a wash with FACs buffer (1× PBS containing 0.5% bovine serum albumin and 1mM EDTA) and incubation with a saturating solution of Fc block (BD Biosciences, San Jose, CA, USA), cells were stained for surface antigens for 25 min in the dark on ice. Antibodies against mouse CD11b, CD45, MHC-II, and CD31 were all purchased from Thermo Fisher Scientific, MA, USA and used as 0.125—0.5μg/10^6^ cells, as recommended by the manufacturer. Cells were examined for intracellular levels of various proinflammatory factors: TNFa, CCL2 and inducible nitric oxide synthase (iNOS). For intracellular staining, cells were fixed with fixation buffer and then permeabilized using intracellular fixation and permeabilization buffer(eBioscience, USA). Cells were then stained with fluorochrome conjugated-antibodies for the intracellular immune factors for another 1 h at room temperature in the dark. After another wash with 1x permeabilization buffer, cells were resuspended in 250–300μl FACs buffer and immediately quantified in the flow cytometer. At least 50,000 live cell events were collected for each sample. Single-stained controls and isotype controls were used for laser compensation and data analysis. Data were acquired using the BD LSR Fortessa cell analyzer (BD Biosciences, San Jose, CA) and analyzed using Flow Jo software v10.7.1.

### Flow cytometry strategy gating

The gating strategy for determining different cell subsets in the brain tissues is similar to that described in our prior reports[6, 24]. Briefly, doublets (cell clumps) were excluded, and the live cells were identified based on their size, granularity (FSC v SSC) and negative viability dye staining. Cerebrovascular endothelial cells were identified based on negative expression of the common leukocyte marker, CD45 and positive staining for PECAM-1 (CD31, Platelet endothelial cell adhesion molecule-1). All CD45 + cells were first gated for peripheral mononuclear neutrophil (PMN) or neutrophil marker, Ly6G (1A8) staining, which were also verified by their positive CD11b staining. The population of CD45 + 1A8–(leukocytes that are not neutrophils) was further analyzed to identify microglia with low or medium expression of CD45 (CD45 low/med, CD11b+) as distinguished from infiltrating macrophages/monocytes with CD45high expression (CD45high CD11b+). Activated microglia were distinguished from non-activated microglia based on combined expression levels of CD45 and CD11b[25]. Additionally, infiltrating monocytes/macrophages were further analyzed for Ly6C expression to identify inflammatory monocytes (1A8–CD11b + CD45high Ly6C+). Median or geometric mean fluorescent intensities were plotted for activation markers or cytokine expression on these different immune and endothelial cell subsets.

### Metabolomics tissue preparation and analysis

Briefly, each hippocampus sample (~ 20 mg, n = 6) was homogenized in 200 μL MeOH:PBS (4:1, v:v, containing 1,810.5 μM ^13^C_3_-lactate and 142 μM ^13^ 3 C_5_-glutamic Acid) in an Eppendorf tube using a Bullet Blender homogenizer (Next Advance, Averill Park, NY). Then 800 μL MeOH:PBS (4:1, v:v, containing 1,810.5 μM ^13^C_3_-lactate and 142 μM ^13^C_5_-glutamic Acid) was added and, after vortexing for 10 s, the samples were stored at −20°C for 30 min. The samples were then sonicated in an ice bath for 30 min. The samples were centrifuged at 14,000 RPM for 10 min (4°C), and 800 μL of supernatant was transferred to a new Eppendorf tube. The samples were then dried under vacuum using a CentriVap Concentrator (Labconco, Fort Scott, KS). Prior to mass spectrometry analysis, the obtained residue was reconstituted in 150 μL 40% PBS/60% acetonitrile. A quality control sample was pooled from all the study samples.

Targeted liquid chromatography–tandem mass spectrometry (LC–MS/MS) technique was used to measure NAD^+^ metabolites, and it is similar to several recent reports (Carroll et al. 2015; Eghlimi et al. 2020; Gu et al. 2016; Gu et al. 2015; Jasbi et al. 2019; Shi et al. 2019). Briefly, LC-MS/MS experiments were performed on an Agilent 1290 UPLC-6490 QQQ-MS (Santa Clara, CA) system. Each hippocampus sample was injected twice: first a 10 μL volume for analysis using negative ionization mode and second a 4 μL volume for analysis using positive ionization mode. Both chromatographic separations were performed in hydrophilic interaction chromatography mode on a Waters XBridge BEH Amide column (150 × 2.1 mm, 2.5 μm particle size, Waters Corporation, Milford, MA). The flow rate was 0.3 mL/min, auto-sampler temperature was kept at 4°C, and the column compartment was set at 40°C. The mobile phase was composed of Solvents A (10 mM ammonium acetate, 10 mM ammonium hydroxide in 95% H_2_O/5% acetonitrile) and B (10 mM ammonium acetate, 10 mM ammonium hydroxide in 95% acetonitrile/5% H_2_O). After the initial 1 min isocratic elution of 90% B, the percentage of Solvent B decreased to 40% at t = 11 min. The composition of Solvent B maintained at 40% for 4 min (t = 15 min), and then the percentage of B gradually went back to 90%, to prepare for the next injection. The mass spectrometer is equipped with an electrospray ionization source. Targeted data acquisition was performed in multiple-reaction-monitoring mode. The whole LC-MS system was controlled by Agilent Masshunter Workstation software (Santa Clara, CA). The extracted MRM peaks were integrated using Agilent MassHunter Quantitative Data Analysis (Santa Clara, CA). Resultant data were normalized by tissue weight before subsequent normalization steps.

The untargeted LC-MS metabolomics method used here was modeled after that developed and used in a growing number of studies[26–29]. Briefly, all LC-MS experiments were performed on a Thermo Vanquish UPLC-Exploris 240 Orbitrap MS instrument (Waltham, MA). The LC conditions were the same as those in targeted metabolomics. Using mass spectrometer equipped with an electrospray ionization (ESI) source, we will collect untargeted data from 70 to 1050 m/z. To identify peaks from the MS spectra, we made extensive use of the in-house chemical standards (~ 600 aqueous metabolites), and in addition, we searched the resulting MS spectra against the HMDB library, Lipidmap database, METLIN database, as well as commercial databases including mzCloud, Metabolika, and ChemSpider. The absolute intensity threshold for the MS data extraction was 1,000, and the mass accuracy limit was set to 5 ppm. Identifications and annotations used available data for retention time (RT), exact mass (MS), MS/MS fragmentation pattern, and isotopic pattern. We used the Thermo Compound Discoverer 3.3 software for aqueous metabolomics data processing. The untargeted data were processed by the software for peak picking, alignment, and normalization. To improve rigor, only the signals/peaks with CV < 20% across quality control (QC) pools, and the signals showing up in > 80% of all the samples were included for further analysis.

### Data analysis and statistics

Quality control samples were inserted at 10-sample intervals during mass spectrometry measurement and were utilized as a pooled sample group to compensate for temporal variability on the machine. Data was analyzed using the R package MetaboAnalyst 5.0[30, 31]. Normality was determined via Shapiro-Wilk testing. In the event of normally distributed data, Student’s t-tests were used. For non-normally distributed data, t-tests were employed on log2() transformed data based on right-tailed distribution. Tests were either conducted in GraphPad Prism v9.1.1, Excel (Version 2211 Build 16.0.15831.20098) or RStudio v1.4.1564, R v4.3.4. Venn diagram was generated using an online multiple list comparison tool[32], with Jaccard ratios calculated via the same software. Student’s t-tests were performed on the datasets and uncorrected as these data were not individually examined, but qualitatively explored as overlapping matrices. These values were input into Excel, RStudio, or downloaded directly for figure generation. Volcano plots were generated using a raw p < 0.05 (NAD^+^ panel; <20 total metabolites) or FDR corrected p < 0.1 (untargeted panel), both with fold-change > 1.5. Heatmapping was performed using limma-based linear regression[33] (untargeted) or interquartile variance (NAD^+^) with metabolite number explicitly stated in the figure legends.

## RESULTS

### Exposure Characterization

8-week-old female C57BL/6J mice were exposed to wood smoke every other day at an average exposure concentration of 0.5mg/m^3^ for two weeks (Fig. 1A–D). Subsequent serial euthanasia occurred at 1-, 3-, 7-, 14-, and 28-days post-exposure (Fig. 1A). Exposure-measured carbon monoxide (CO) and oxides of nitrogen species (NO_X_) were below the USEPA standards (Fig. 1D). Bronchoalveolar lavage samples were taken at each euthanasia timepoint (Fig. 1E) with total cells significantly higher on day 3 post-exposure, but no other differences were seen for other cells (neutrophils, lymphocytes, eosinophils) or on different days. Weight was monitored throughout the study, with no significant separations or deviations observed (Fig. 1F). At euthanasia, brains were rapidly excised and frozen for metabolomics (hippocampus) and flow cytometry (half brain).

### Cerebrovascular Endothelial Cell Neuroinflammatory Activation and Resolution in Response to Woodsmoke

**Figure 2:**
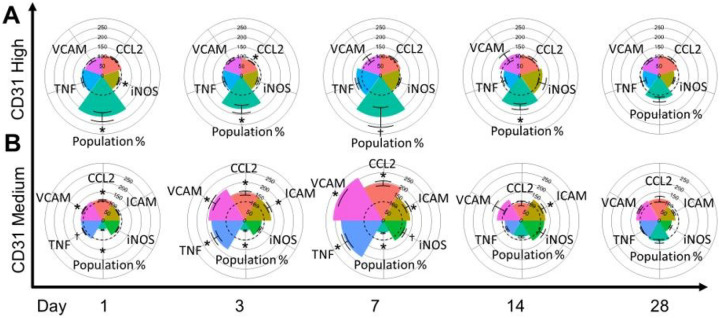
Endothelial Flow Cytometry. (A&B) CD31 (PECAM) endothelial cells representing blood brain barrier; y-axis: marker grouping; x-axis: day post exposure; colored radar plot slice: marker of interest measured; dotted line: 100% baseline control; all values normalized to filtered air (FA) controls. (A) Anti-inflammatory CD31^Hi^ (PECAM^High^) cells showed an immediate increase in population percent and a decrease in iNOS. On day 3, population percent remained significantly increased coinciding with a reduction in CCL2 expression. Population percent remained significantly higher on days 7 and 14. (B) Proinflammatory CD31^Med^ (PECAM^Med^) saw a decreased population percent, but increased TNF, VCAM-1, and CCL2 expression through days 1–7. ICAM expression increased on day 3 and remained up until day 14.

A list of p-values can be found in Table 1. After gating, we observed two distinct populations of endothelial cells expressing a range of PECAM (Fig. 2), consistent with our previous study of real-world wildfire smoke[6]. The population of PECAM^High^ was significantly increased from day 1 (D1) post-exposure until day 14 (D14; Fig. 2A). The percentage of cells on each day relative to total cells is as follows (D[x^marker^]: FA%/WS%). D1^PECAMHigh^:1.64/3.05, D1^PECAMMed^:17.73/8.30, D3^PECAMHigh^:2.28/3.99, D3^PECAMMed^:3.09/1.58, D7^PECAMHigh^: 0.41/0.86, D7^PECAMMed^: 3.88/2.4525, D14^PECAMHigh^:1.45/2.47, D14^PECAMMed^:4.60/3.99, D28^PECAMHigh^:2.16/2.65, D28^PECAMMed^:14.53/15.63. The population of endothelial cells expressing PECAM^High^ has been suggested as an anti-inflammatory reaction to stimuli[16, 34, 35], which is supported by the significant decreases in iNOS and CCL2 expression on D1 and D3, respectively. The proinflammatory population of PECAM^Med^ was depleted until D14, which then returned to normal levels relative to control. Although PECAM^Med^ was reduced in number, an inflammatory phenotype was generated(Fig. 2B), with marked increases to VCAM-1, TNFα, and CCL2 on D1, an additional significant increase to ICAM-1 on D3, with a further increase to iNOS on D7. By D14, all markers except ICAM-1 reduced to normal levels relative to control. By D28, all markers measured returned to baseline expression levels. Taken together, these data indicate that a small population of endothelial cells are responsible for the bulk of signaling and the proinflammatory response associated with our biomass exposure, and that endothelial cells are dynamically changing their phenotype following neuroimmune insult from woodsmoke inhalation.

### Immune Cell Infiltration in the Brain and Resolution in Response to Woodsmoke

**Figure 3:**
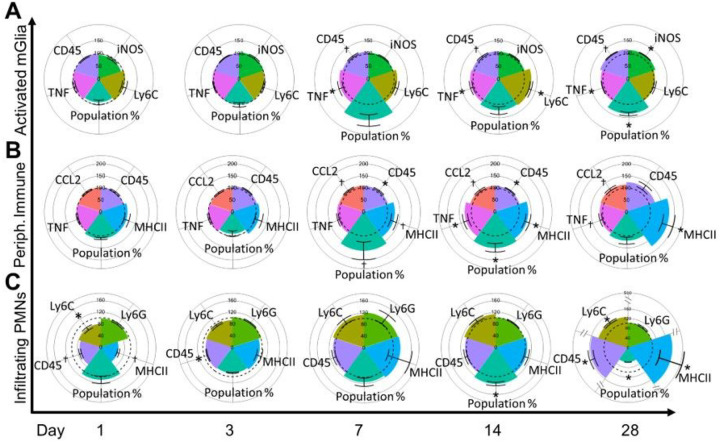
Microglial Activation and Peripheral Immune Infiltration. (A-C) y-axis: cell subset grouping; x-axis: day post exposure; colored radar plot slice: marker of interest measured; dotted line: 100% baseline control; all values normalized to filtered air (FA) controls. (A) Activated microglial population increased on day 28. TNF increased on day 7 and remained increased at day 28. CD45 trended upward stating on day 7 through day 28. iNOS expression increased on day 28. (B) Nonspecific peripheral immune infiltration population percent trended upward on day 7, showed significant increases on day 14, and was resolved by day 28. TNF expression increased on day 14 and trended upward on day 28. CCL2 expression trended upward starting on day 7 and remained trending upward on day 28. CD45 expression increased on day 7 and 14 but was resolved by day 28. MHCII trended upward on day 7 and showed significant increases on days 14–28. (C) Infiltrating polymorphonuclear neutrophils (PMNs) showed a reduction in Ly6C on day 1 with a trending decrease to MHCII. CD45 trended downward on day 1 and was significantly reduced on day 3. On day 14, population percent increased. On day 28, population percent decreased, but CD45, Ly6C, and MHCII showed significant increases. * p<0.05, † p<0.10.

The adhesion molecule results in endothelial cells prompted a thorough investigation of microglial activation and peripheral immune infiltrates in the brain, which included neutrophils and nonspecific peripheral immune cell infiltration (Fig. 3). This investigation revealed no statistically significant increase in the percentae of activated microglia until 28 days post-exposure. However, activated microglia increased expression of TNFα and CD45 beginning on D7, which persisted until D28 post-exposure (Fig. 3A, Table 1). Furthermore, increased expression of Ly6C and iNOS were observed on days 14 and 28, respectively. When examining non-specific peripheral immune infiltrates (Fig. 3B, Table 1), we observed trending increases to activated microglia population percent beginning on D7, which coincided with increased expression of MHCII, CCL2, and CD45. All markers were either significantly increased or trending on D14, with MHCII significantly increased on D28, along with trending increases to CCL2 and TNFα. When examining neutrophil infiltration (Fig. 3C), we observed reduced expression on D1 of CD45, Ly6C and MHCII, with CD45 remaining depressed on D3. On D14, a significant population increase was observed. Interestingly, D28 post-exposure saw a significant reduction in population percent, coinciding with an increase in CD45, Ly6C, and MHCII. These data largely show an initial reduction in neutrophils after exposure, a neutrophil population increase on D14, with subsequent neutrophil exclusion on 28. However, the neutrophils remaining within the hippocampus on D28 appeared to be highly inflammatory with significantly elevated CD45, Ly6C, and MHCII. Taken together, these data indicated a long-term (at least 28-day) microglial response, a D14 neutrophil and non-specific peripheral immune infiltration, with a small, but potent inflammatory neutrophil population still present on D28 (Supp. Figure 1).

### Metabolomic Changes in the Brain following Wood Smoke Exposure

The dynamic neurocellular alterations following woodsmoke inhalation prompted an investigation into small molecule changes in the hippocampal region. We utilized both an untargeted metabolomic panel and a panel targeted to NAD^+^ synthesis pathways. Broadly, the targeted NAD^+^ panel was utilized based on our group’s previous work showing woodsmoke-induced decreases in neuroprotective metabolites and decreases to NAD^+^, itself[6].

**Figure 4:**
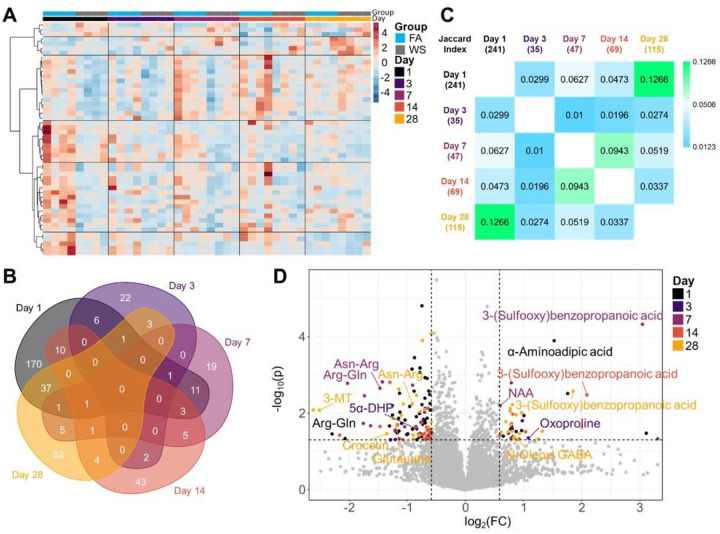
Untargeted Metabolomic Temporal Analysis. (A) Heatmap of top 50 significant metabolites. Limma-based linear modeling, Pearson distance, Ward clustering. (B) Venn diagram of statistically significant metabolites per day. Day 1 has the strongest overlapping signature with each subsequent day. (C) Jaccard index correction for multiple statistically significant metabolites. Day 1 has the strongest overlap with day 28, while days 7 and 14 have the strongest overlap with each other. (B & C) Data normalized by quality control, unlogged, student’s t-test. (D) Volcano plot overlay from each day. Abbreviations: Arg: Arginine, Asn: Asparagine, DHP: Dihydroprogesterone, Gln: glutamine, MT: Methoxytyramine, NAA: N-acetyl-L-aspartic acid. Data normalized by quality control samples and log_2_().

Regarding the untargeted panel, initial heatmapping revealed consistent visual patterns that persisted over the 28-day time course (Fig. 4A). This method utilized a limma-based linear modeling to show 113 total significantly altered metabolites (Supplementary Table 1). However, statistical significance alone gave no indicator of overlapping of significant metabolites between each day measured. Thus, we utilized a Venn diagram to illustrate significantly altered metabolites that overlapped per day (Fig. 4B). These data showed only minor overlaps, initially indicating temporal compensation mechanisms of stimuli and response. Broadly, D1 showed the largest overlap with each subsequent day (D3 = 6, D7 = 11, D14 = 10, and D28 = 37 metabolites). However, we reasoned the sheer number of statistically significant metabolites from D1 could be casting the widest net to overlap other days. From this, we employed Jaccard calculations that reduce numerical weight bias (Fig. 4C). Regardless of methodology employed, we see the strongest correlation between days 1 & 28 (J-index = 0.1266). Interestingly, these data showed a strong initial hippocampal impact of 241 significantly altered metabolites on D1, rapid compensation and decrease to a mere 35 significant metabolites on D3, with a gradual ramping up of 47 metabolites on D7, 69 on D14, and increasing to 115 significantly altered metabolites on D28 (Supp. Figure 1). These data mirror the dynamic pattern seen in the cell populations. Namely, a large initial response was also seen in the endothelial phenotype, while peripheral immune infiltration began subsequently, resulting in an inflammatory response that was not fully resolved by D28. The global metabolomic effect may be influenced by the specific and complex cellular responses resulting from activation and suppression of cell populations like the endothelium, resident neuro-immune cells, different neuronal subtypes, and infiltrating peripheral immune cells. More specifically, the endothelial response on D1 appears to cause the greatest shift in significantly altered metabolites, while the gradual increase of immune responses caused a gradual increase in significantly altered metabolites.

To examine individual metabolites affected, we overlapped volcano plots for each day (Fig. 4D). On D1, we observed an increase to α-aminoadipic acid, which has been implicated as an inhibitory regulator of kynurenic acid synthesis in the hippocampus[36]. Additionally, we observed a decrease to arginine-glutamine (Arg-Gln), and a potential compensation mechanism in the D3 increase to oxoproline — a facilitator of amino acid transport across the BBB[37]. Of note, we observed a reduction in the 5α-Pregnane-3,20-dione (5α-Dihydroprogesterone; 5α-DHP), and a D7 decreased abundance of the dipeptides arg-gln and asn-arg (asparagine-arginine). Additionally, we observed an increased level of N-acetyl-L-aspartic acid (NAA), which is known to have a positive impact on working memory[38]; perhaps a compensation mechanism from downregulated metabolites seen on days 1–3. On D28, notable observations included a decrease in 3-methoxytyramine (3-MT) and glutaurine. Taken together, these data potentially indicate a consistent reduction in amino acid dipeptides over time and suggest mood alterations.

**Figure 5:**
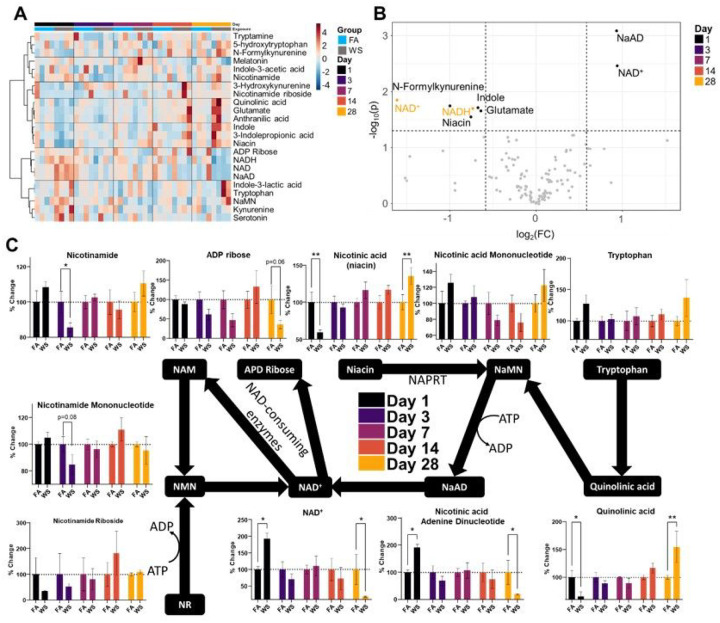
Targeted NAD+ Metabolomic Temporal Analysis. (A) Heatmap of all metabolites within the panel. Interquartile range variance shown, Pearson distance, Ward clustering. (B) Volcano plot overlay from each day. Abbreviations: NaAD: Nicotinic acid Adenine Dinucleotide, NAD: Nicotinamide Adenine Dinucleotide, NADH: Nicotinamide Adenine Dinucleotide + Hydrogen. Data normalized by quality control samples, log_2_() transformed. (C) Preiss–Handler and salvage pathways with accompanying temporal bar charts. Data normalized by quality control samples, log_2_() transformed and outliers removed.

From the NAD^+^ panel, initial heatmapping revealed less consistent patterns than the untargeted panel (Fig. 5A). Furthermore, only 2 woodsmoke-responsive metabolites were deemed significant in the exposed group after limma-based linear modeling (NADH and Indole, data not shown) resulting in the variance-based interquartile range being represented (Fig. 5A). Through similar volcano plot overlaying as in Fig. 3D, we overlayed all days of our NAD^+^ panel on a single plot (Fig. 5A). Largely, D1 revealed that NAD^+^ and Nicotinic acid adenine dinucleotide (NaAD) abundances were increased, with decreased abundance seen in glutamate, indole, niacin, and N-formylkynurenine. Although no metabolites appeared significantly altered by woodsmoke between D3–D14, D28 metabolites of NAD^+^ and NADH showed decreased abundance. After removing outliers, we plotted graphs on the Preiss–Handler and salvage pathways to examine specific points of interest for future investigation (Fig. 5C). Quinolinic acid and niacin were immediately decreased on D1, but slowly recovered and overshot baseline levels on D28. Nicotinic acid adenine dinucleotide (NaAD) and NAD^+^ levels followed an opposite pattern of increased abundance on D1 followed by eventual deficits on D28. The precursor nicotinamide mononucleotide exhibited no such trend, nor did the post-cursors nicotinamide or ADP ribose. Taken together, these data indicate trends for the hippocampus that persist for the duration of our 28-day time course. Furthermore, the NAD^+^ depletion is particularly unsettling and warrants further investigation as the precursors and post-cursors do not indicate the reason for abundance and depletion seen in this study.

## DISCUSSION

Summarily, these data indicate that the neuroinflammatory and neurometabolomic alterations following woodsmoke exposure can persist up to and possibly longer than 28 days. The neuroinflammation driven by woodsmoke was consistent with that observed after a real-world exposure to PM in New Mexico that was largely derived from California wildfires[6]. In the present study, fresh biomass combustion emissions were used, at a higher overall 24h average concentration than the real-world exposure (44 vs 18 μg/m^3^)[39]. The observation of a prolonged period of neuroinflammation and hippocampal metabolic alterations out to 28 days after the end of exposures aligns with epidemiological findings of persistent deficits in cognition and attention in woodsmoke-exposed populations[7], as well as prolonged post-traumatic stress and depression that accompany severe wildfire events[40–43].

Neurovascular endothelial cells exhibited a response to woodsmoke that included both inflammatory and anti-inflammatory components. Following two weeks of intermittent exposure, mice showed a robust increase in the PECAM^High^ endothelial cells that exhibited reduced relative expression of inflammatory markers (CCL2, iNOS). We observed a reduced population of PECAM^Med^ endothelial cells associated with promoting the neuroinflammatory response to woodsmoke, but these cells exhibited increased expression of VCAM-1, ICAM-1, CCL2, TNFa, and iNOS to varying degrees even 14 days after the exposures had ended. Both pro- and anti-inflammatory endothelial cell responses had largely subsided by day 28. Current understanding points to a reduced endothelial expression of PECAM-1 in Alzheimer’s disease compared to healthy individuals and decreased expression in arterial beds relative to venous and capillary[44]. Accelerating recovery of inflammation may have value for neurological health, in which case both populations of anti-inflammatory and proinflammatory endothelial cells represent interesting therapeutic targets after woodsmoke exposure.

Interestingly, the activated microglial response to woodsmoke increased on D7, continuing through D28. Activated microglia can release quinolinic acid, and our metabolomic results show a gradual increasing concentration of quinolinic acid until statistical significance is achieved on D28. If this response lasts, neurological damage could occur due to quinolinic acid having the potential for excitotoxic effects. Here we reported a population of activated microglia expressing Ly6C on D14. However, there is evidence that these could be microglial precursors being differentiated and recruited to fight ongoing infections/stimuli[20]. If true, this would represent a first wave elimination of microglia, perhaps after phagocytosing or through other elimination pathways. These would necessarily need to be replaced through the differentiation of microglial precursors and recruited to the site of ongoing response(s). The subsequent D28 increase to activated microglia population percent could be confirming the current literature and the hypothesis posited here.

We also observed a D14 neutrophil and non-specific peripheral immune invasion. On this point, there are two current schools of thought, with one postulate that the inhaled particles are able to escape the lungs and enter the circulation[45,46]. Upon reaching the BBB, the peripheral immune system is notified and recruited for a response to the inhaled particles. Another school of thought is that the inhaled particles cause a localized response in the lung, and the fragmented response peptides escape into the circulation from the lungs[13,47,48]. When the fragmented lung response peptides reach the BBB, the peripheral immune system is notified and recruited for a response to the self-peptides. Although the molecule/particle causing the recruitment will change based on the theory, the end result for immune recruitment holds based on our peripheral immune response. This conclusion is also strengthened based on the constant and large number of neutrophils in circulation[49,50]. Additionally, ozone inhalation can activate microglia and cause BBB decrements[10,24], despite being so reactive that it is unable to penetrate further than 0.1 μm in the epithelial fluid lining of the lung[51,52]. A final thought regarding neutrophils, the remaining neutrophil population on D28 was significantly lower than FA controls but appeared to be responding strongly to a stimulus. Conceivably this could reflect a penetrance of particulates into the brain and an immunological process for removal.

Hippocampal metabolite changes may reaffirm recent observations of reduced learning and attention after wildfire exposure, using an app-based experiment performed in humans[7]. This conclusion is based on our observed reductions in glutamate, glutaurine, 3-MT, 5α-DHP, NAD^+^, and the decreased ratio of NAD^+^/NADH. 5α-DHP is a neuroprotective[53] agonist for the GABA_A_ receptor that is synthesized from progesterone and reductions have been linked to social isolation and depression[54]. 5α-DHP has high affinity for the progesterone receptor (which are found in the hippocampus) and regulates DNA transcription[54]. 3-MT is a dopamine metabolite implicated in movement control[55], while glutaurine naturally mimics the effects of valium[56]. Broadly, we observed the strongest metabolic response on D1, with an immediate reduction in response by D3, and a progressive return to strong response by D28. These data largely agree with the flow cytometry results that indicate a delayed peripheral immune response and microglial activation persisting through D28. Our intent for this study was to reveal the time course for resolution of the neurological effects of woodsmoke exposure; 28 days was seemingly insufficient in this mouse model.

Through the untargeted panel, we observed an increase in α-Aminoadipic acid on D1 (Fig. 4D), which has been shown to downregulate kynurenic acid[36]. This increase coincided with decreases in quinolinic acid and N-Formylkynurenine abundances seen in our NAD^+^ panel (Fig. 5B, C). Together, these could indicate that the kynurenine and tryptophan pathways were shifted toward NAD^+^ production, rather than production of kynurenic acid and quinolinic acid[57]. This is based on α-Aminoadipic acid downregulating kynurenic acid, which implicates the shift toward quinolinic acid and NAD^+^ production. This is further corroborated by the D1 decrease in niacin (Fig. 5C) and increased NAD^+^ abundance (Fig. 5C). Quinolinic acid can be excitotoxic with persistent exposure through activated microglia[58]. However, kynurenic acid is released from astrocytes and promotes neuronal stability[59,60]. The increased abundance of α-Aminoadipic acid and reductions downregulation of kynurenic acid, along with reductions in 3-MT and 5α-DHP may contribute to the epidemiological findings of persistent deficits in cognition, attention and mood alterations observed following woodsmoke exposure[54,55].

Additional interesting and confounding points related to hippocampal metabolite changes include the consistent reductions in amino acid dipeptides over time and increases to 3-(sulfooxy) benzenepropanoic acid. We are yet unclear as to the meaning, which warrants further investigation. Additionally, we observed a decrease to the dipeptide arginine-glutamine (Arg-Gln), which is formed from L-arginyl and L-glutamine residues. On D3, we observed a potential compensation mechanism for reduced Arg-Gln through an increased oxoproline. This metabolite has been shown to facilitate amino acid transport across the blood brain barrier[37]. Finally, D28 crocetin was decreased and has been shown to protect against beta-amyloid brain abundance[61]. Additionally, 3-O-beta-D-glactosyl-sn-glycerol abundance was decreased, whose biosynthesis have been observed in mouse brain microsomes[62], with implications in anti-inflammatory potential[63]. The observed increase to N-Oleoyl GABA will mimic GABA effects and reduce neuronal excitability and inhibition of neurotransmission. Curiously, 3-(sulfooxy)benzenepropanoic acid was observed to be increased on D7, D14, and D28; however, the neurological functions are unknown and the proposed source is food consumption. Yet, the mouse weights did not statistically deviate from FA controls throughout the course of experiments (Supplementary Figure 1).

Regarding the NAD^+^ panel, the decreased abundance at D28 may imply increased demand from NAD^+^ consuming enzymes or pathways. Although we were unable to determine which enzyme or pathway was consuming the NAD^+^, these data do corroborate previous studies that show a decrease in NAD^+^ abundance after wildfire smoke exposure[6]. There are many important NAD^+^ consuming enzymes within the brain parenchyma, including sirtuins and poly(ADP-ribose) polymerases. Without these molecules — and coupled with the highly responsive neutrophil data on D28 — there exists increased potential for damage and inflammatory persistence. Furthermore, if the model of fragmented peptides reaching the BBB is correct, then these neutrophils are potentially reacting to self. This has much broader implications, given that wildfire acres burned yearly have roughly doubled since 1985 and the steadily increasing incidence of autoimmunity based on epidemiological evidence[1,64].

Caveats to this study include a limited study design related to fuel source, timing of exposures, mouse model (age and sex), duration of examined timepoints, and observational outcomes. There are a multitude of organic and non-organic fuel sources for wildfires, including brush, grasses, leaves, cars, paint, asbestos, plastics, and others. Additionally, our exposure paradigm utilized replicability at the partial expense of real-world parameters. Our exposure levels were quite modest compared to some wildfires, including reported exposures in Washington State from 2020, where it was estimated that 7.1 million people were exposed to average daily particulate matter concentrations ranging from 50 – 150 μg/m^3^ for 13 consecutive days[39]. We chose an intermittent exposure scenario, however, as during wildfire events, the ambient particulate matter concentration can fluctuate significantly between days, or even hours as prevailing winds change direction. Finally, the descriptive nature and timepoints utilized did not allow for a full resolution of immune inflammation, nor did we explore behavioral or histopathological impacts of the major findings.

Summarily, the major findings of this study include the duration of resolution, impacts on inflammatory and metabolite profiles, and corroboration with recent human exposure data. The duration of endothelial neuroimmune activity and resolution appeared to be resolved within 7–14 days. However, microglial activation, peripheral immune infiltration, and neutrophil invasion began during endothelial response and lasted until our final timepoint examined (Supplemental Figure 2). Metabolomic profiles saw the largest effect on D1, which is likely to be associated with endothelial response. This decreased by day 3 but gradually ramped up to nearly half the original response by day 28, which is likely to be associated with the immune cell response. Future studies could employ vulnerable models to delineate this endothelial cell-specific response versus immune response and increase resolution of neuroimmune vs metabolomic changes per cell type to understand the relationships. Finally, implied behavioral, cognitive, and mood alterations could be revisited to determine mechanistic alterations and confirm observational result.

## Figures and Tables

**Table 1.** P-values calculated from flow cytometry results. Yellow: trending increase/decrease. Green, significant difference.

	Day					
	Marker	1	3	7	14	28
**PECAM High**	**Population**	0.014	0.025	0.108	0.015	0.173
	**TNF**	0.673	0.373	0.077	0.197	0.344
	**VCAM-1**	0.481	0.865	0.500	0.357	0.337
	**CCL2**	0.181	0.040	0.102	0.448	0.965
	**iNOS**	0.022	0.158	0.125	0.301	0.396
**PECAM Medium**	**Population**	0.015	0.002	0.006	0.468	0.568
	**TNF**	0.060	0.003	0.000	0.157	0.948
	**VCAM-1**	0.043	0.002	0.001	0.415	0.150
	**CCL2**	0.019	0.003	0.000	0.552	0.348
	**ICAM**	0.078	0.010	0.009	0.021	0.723
	**iNOS**	0.103	0.158	0.048	0.328	0.834
**Activated Microglia**	**Population**	0.548	0.958	0.103	0.427	0.017
	**TNF**	0.595	0.066	0.036	0.043	0.018
	**CD45**	0.512	0.980	0.065	0.094	0.099
	**iNOS**	0.150	0.705	0.630	0.116	0.047
	**Ly6C**	0,854	0.657	0.094	0.013	0.185
**Peripheral Immune Infiltrates**	**Population**	0.013	0.222	0.104	0.042	0.227
	**TNF**	0.545	0.627	0.153	0.016	0.104
	**CCL2**	0.564	0.083	0.102	0.091	0.085
	**CD45**	0.931	0.166	0.040	0.028	0.138
	**MHCII**	0.462	0.209	0.106	0.037	0.035
**Infiltrating Neutrophils**	**Population**	0.501	0.328	0.448	0.046	0.041
	**CD45**	0.060	0.004	0.495	0.447	0.031
	**Ly6C**	0.023	0.079	0.448	0.300	0.004
	**Ly6G**	0.839	0.414	0.369	0.749	0.914
	**MHCII**	0.079	0.441	0.463	0.592	0.053

## Data Availability

All data are available through the corresponding author upon request.
